# Gestational Hydronephrosis: A Retrospective Analysis of the Clinical Outcomes of Ureteral Stent Placement Versus Conservative Treatment

**DOI:** 10.3390/medicina61050845

**Published:** 2025-05-04

**Authors:** Dursun Baba, Engin Yurtçu, Burak Ayvacık, Yusuf Salih Küçük, Arda Taşkın Taşkıran, Mehmet Ali Özel, Ahmet Yıldırım Balık, Ekrem Başaran, Betül Keyif

**Affiliations:** 1 Department of Urology, Faculty of Medicine, Duzce University, Duzce 81100, Türkiye; burakayvacik@duzce.edu.tr (B.A.); yusufsalihkucuk@duzce.edu.tr (Y.S.K.); ardataskiran@duzce.edu.tr (A.T.T.); ahmetbalik@duzce.edu.tr (A.Y.B.); ekrembasaran@duzce.edu.tr (E.B.); 2 Department of Obstetrics and Gynecology, Faculty of Medicine, Duzce University, Duzce 81100, Türkiye; enginyurtcu@duzce.edu.tr (E.Y.); betulkeyif@duzce.edu.tr (B.K.); 3 Department of Radiology, Faculty of Medicine, Duzce University, Duzce 81100, Türkiye; mehmetaliozel@duzce.edu.tr

**Keywords:** Apgar score, gestational diabetes, gestational hydronephrosis, obstetric outcomes, renal function, ureteral stent

## Abstract

*Background and Objectives:* Gestational hydronephrosis (GH) is a physiological condition commonly observed during pregnancy, resulting from hormonal effects and mechanical compression of the ureters by the enlarging uterus. Although often asymptomatic, GH can cause urinary stasis, recurrent infections, and renal function impairment in symptomatic cases. The optimal management of such cases remains controversial, especially regarding the role of ureteral stent placement. This study aimed to compare clinical outcomes—including renal function, inflammatory markers, and obstetric parameters—in pregnant women with symptomatic GH who underwent ureteral stent placement versus those managed conservatively. *Materials and Methods:* We conducted a retrospective cohort study at Düzce University Hospital between 2020 and 2024, including 40 pregnant women diagnosed with symptomatic GH. The patients were divided into the following two groups: those who received a ureteral stent (*n* = 20) and those who were managed with conservative treatment (*n* = 20). Conservative management included hydration therapy, acetaminophen-based analgesia, and close clinical monitoring. The parameters assessed included serum creatinine, estimated glomerular filtration rate (GFR), inflammatory markers (C-reactive protein, erythrocyte sedimentation rate, and white blood cell count), urinary findings, obstetric outcomes, and postpartum complications. Statistical significance was set at *p* < 0.05. *Results:* Gestational age at diagnosis was significantly higher in the stent group (29.1 ± 3.2 weeks) than in the non-stent group (27.1 ± 3.5 weeks; *p* = 0.045), possibly reflecting increased mechanical compression in later pregnancy. Renal function parameters (serum creatinine and GFR), inflammatory markers (CRP, ESR, and WBC count), and obstetric outcomes (birth weight, Apgar scores) showed no significant differences between groups (*p* > 0.05). Interestingly, gestational diabetes mellitus (GDM) was more prevalent in the non-stent group (20% vs. 5%; *p* = 0.042), although no significant differences were found in fasting glucose levels. *Conclusions:* Ureteral stent placement in symptomatic GH does not appear to significantly improve renal function or obstetric outcomes. However, it may provide symptom relief in select patients with persistent or severe discomfort. Given the limitations of retrospective data and a small sample size, further prospective studies with larger cohorts and quality-of-life assessments are warranted to optimize management strategies and enhance patient-centered care.

## 1. Introduction

Pregnancy induces profound physiological and anatomical changes in the urinary system, significantly affecting renal hemodynamics and urinary drainage. One of the most common urological manifestations is gestational hydronephrosis (GH), typically resulting from mechanical compression of the ureters by the enlarging uterus combined with hormonal effects—most notably the smooth muscle relaxation induced by progesterone [1,2]. While often considered a physiological adaptation, GH may become clinically significant in symptomatic cases, particularly during the third trimester when uterine size and fetal weight are maximal. It predominantly affects the right ureter, owing to the anatomical positioning of the uterus and adjacent vasculature [1,3].

Although usually benign and self-limiting, GH may lead to urinary stasis, recurrent urinary tract infections (UTIs), renal function impairment, and—in severe cases—acute pyelonephritis [4,5]. Timely diagnosis and individualized management are therefore essential for protecting maternal and fetal health. Nevertheless, there is no universally accepted clinical guideline for the management of symptomatic GH. While many clinicians prefer conservative treatment with hydration, analgesics, and close monitoring, others recommend ureteral double-J (DJ) stent placement for patients with persistent symptoms, suspected renal compromise, or recurrent infections [3,6,7].

The clinical utility of ureteral stents during pregnancy remains debated. Although stents ensure ureteral drainage and may help preserve the glomerular filtration rate (GFR), they are also associated with discomfort, hematuria, stent migration, infection, and encrustation—factors that may negatively impact quality of life [2,8,9,10]. Moreover, their influence on broader obstetric outcomes—including gestational diabetes mellitus (GDM), hypertensive disorders, and neonatal morbidity—remains insufficiently characterized.

In this context, our study aims to compare the clinical outcomes of pregnant women with symptomatic GH who were treated either with ureteral stent placement or managed conservatively. Specifically, we sought to evaluate differences in renal function (creatinine, GFR), inflammatory markers, obstetric outcomes, and postpartum complications. Additionally, we aimed to assess whether patient selection, based on symptom severity and gestational stage, could inform a more risk-based and patient-centered approach to management. This work contributes to institutional experience and provides a basis for future prospective studies investigating optimal care strategies for GH.

## 2. Materials and Methods

### 2.1. Study Design and Patient Selection

This retrospective cohort study was approved by the Ethics Committee of Düzce University Faculty of Medicine (Approval No: 2025/52, Date: 24 February 2025) and conducted in accordance with the Declaration of Helsinki. The study included pregnant women diagnosed with symptomatic gestational hydronephrosis (GH) who were followed at the Gynecology and Obstetrics and Urology Clinics of Düzce University Hospital between 2020 and 2024. The patients were divided into the following two groups based on treatment modality: those who underwent ureteral stent placement (stent group) and those who received conservative treatment (non-stent group).

### 2.2. Inclusion and Exclusion Criteria

The inclusion criteria were as follows: a clinically and radiologically confirmed diagnosis of symptomatic GH, singleton pregnancy, and availability of complete medical records. Symptomatic GH was defined by the presence of symptoms such as flank pain, recurrent urinary tract infections, or renal dysfunction. The exclusion criteria included pre-existing urological disease (e.g., nephrolithiasis, congenital anomalies, and ureteropelvic junction obstruction), chronic renal failure, multiple pregnancy, and incomplete clinical data. Patients with primary ureteral calculi or anatomical obstruction as the underlying cause of hydronephrosis were also excluded.

### 2.3. Diagnostic Evaluation and Treatment Selection

The diagnosis of gestational hydronephrosis was based on clinical findings, laboratory tests, and ultrasonographic (USG) imaging. The severity of hydronephrosis was assessed according to the anteroposterior (AP) diameter of the renal pelvis, with mild hydronephrosis defined as an AP diameter less than 10 mm, moderate as 10–15 mm, marked as 15–20 mm, and severe as greater than 20 mm [11]. Conservative treatment included hydration therapy, acetaminophen-based analgesia, and regular clinical monitoring. Indications for stent placement included persistent severe flank pain despite analgesia, recurrent urinary infections defined as three or more UTI episodes, or signs of renal impairment indicated by a GFR less than 60 mL/min or a serum creatinine level above 1.5 mg/dL [11].

### 2.4. Surgical Procedure—Ureteral Stent Placement

All ureteral double-J stent procedures were performed by experienced urologists under sterile conditions using either rigid or flexible cystoscopy with a pregnancy-appropriate anesthesia protocol. A 5–6 French ureteral stent was placed retrogradely under guidewire guidance. Stent placement was confirmed by ultrasonography or intraoperative fluoroscopy. Urine cultures were obtained, and prophylactic antibiotic therapy was administered in all cases. Patients were monitored with regular laboratory tests and ultrasonographic evaluations during the follow-up, and the stents were typically removed within six weeks postpartum [12].

### 2.5. Data Collection and Analyzed Parameters

Clinical and laboratory data were retrieved from electronic medical records. The following parameters were recorded and analyzed: maternal age, gestational age at diagnosis, gravida, parity, serum creatinine, estimated glomerular filtration rate (GFR), blood urea nitrogen (BUN), C-reactive protein (CRP), erythrocyte sedimentation rate (ESR), white blood cell (WBC) count, presence of proteinuria, microscopic hematuria, leukocyte esterase on urinalysis, systolic and diastolic blood pressure, anteroposterior renal pelvis diameter, birth weight, Apgar scores at 1 and 5 min, neonatal intensive care unit (NICU) admission, cesarean section, postpartum infection, postpartum hemorrhage, and stent revision. All laboratory and imaging assessments were performed during routine antenatal care or immediately prior to stent placement. Obstetric and neonatal outcomes were obtained from delivery records. It is important to note that BUN was used only as a supportive parameter and not as a diagnostic criterion for renal function.

### 2.6. Statistical Analysis

All statistical analyses were conducted using SPSS version 26.0 (IBM Corp., Armonk, NY, USA). A priori power analysis was performed using G*Power version 3.1.9.7 (Heinrich Heine University, Düsseldorf, Germany), which indicated a required sample size of 18 per group based on an effect size of 0.8, an alpha value of 0.05, and a power of 0.80 [9]. To ensure sufficient power despite potential data loss, a total of 40 participants were included. The distribution of continuous variables was assessed using the Kolmogorov–Smirnov test. Normally distributed variables were analyzed using the independent samples *t*-test, while non-normally distributed data were assessed using the Mann–Whitney U test. Categorical variables were compared using the Chi-square test. Logistic regression analysis was used to identify independent predictors of stent placement, with odds ratios (ORs) and 95% confidence intervals (CIs) reported. Correlation analysis was performed using Pearson’s or Spearman’s correlation coefficients depending on the distribution of the variables. A *p*-value < 0.05 was considered statistically significant.

## 3. Results

The demographic and clinical characteristics of the study population are summarized below. There was no statistically significant difference in maternal age between the stent and non-stent groups (32.3 ± 5.6 vs. 32.5 ± 4.9 years, *p* = 0.754). However, gestational age at the time of GH diagnosis was significantly higher in the stent group (29.1 ± 3.2 weeks) than in the non-stent group (27.1 ± 3.5 weeks, *p* = 0.045), suggesting an increased symptom burden or mechanical pressure with advancing pregnancy.

Renal function parameters, including serum creatinine and estimated glomerular filtration rate (GFR), did not differ significantly between the groups (*p* = 0.612 and *p* = 0.289, respectively). Similarly, inflammatory markers such as C-reactive protein (CRP) and erythrocyte sedimentation rate (ESR) were comparable (*p* = 0.402 and *p* = 0.245). Although not originally reported in Table 1, white blood cell (WBC) count was analyzed and showed no significant difference between the groups (mean WBC count: 10.7 ± 2.4 × 10⁹/L in the stent group vs. 10.2 ± 2.7 × 10⁹/L in the non-stent group, *p* = 0.428).

Urinalysis findings, including proteinuria (25% in the stent group vs. 15% in the non-stent group, *p* = 0.378) and microscopic hematuria (35% vs. 25%, *p* = 0.291), were more frequent in the stent group, but these differences were not statistically significant. Blood pressure values were similar across groups (systolic *p* = 0.458; diastolic *p* = 0.619).

Obstetric outcomes revealed no significant differences in birth weight or Apgar scores. Although the-)mean birth weight was lower in the stent group (2949.2 ± 382.4 g vs. 3069.4 ± 400.2 g), the difference was not statistically significant (*p* = 0.089). Apgar scores at 1 and 5 min were comparable (*p* = 0.254 and *p* = 0.437). Notably, gestational diabetes mellitus (GDM) was significantly more frequent in the non-stent group (20% vs. 5%, *p* = 0.042). However, mean fasting blood glucose levels, which were available for 35 participants, did not significantly differ (mean: 97.8 ± 13.5 mg/dL in the non-stent group vs. 95.4 ± 12.1 mg/dL in the stent group, *p* = 0.231). Glycosuria was inconsistently documented and thus excluded from analysis. The incidence of preeclampsia did not differ significantly between the groups (20% in the stent group vs. 15% in the non-stent group, *p* = 0.285).

Postpartum complications, including infection (15% in the stent group vs. 10% in the non-stent group, *p* = 0.489) and the need for stent revision (5% vs. 0%, *p* = 0.073), were low and did not show statistically significant differences. These findings are detailed in Table 1.

A logistic regression analysis was performed to identify variables that might predict stent placement. As shown in Table 2, none of the examined variables—including maternal age, birth weight, WBC count, hemoglobin levels, and Apgar score at 1 min—were significantly associated with the decision to insert a stent.

Lastly, a correlation analysis was conducted to assess associations between selected clinical variables. As shown in Table 3, no strong correlations were identified. Gestational age showed a weak, non-significant negative correlation with WBC count (r = −0.108, *p* = 0.506), and serum creatinine showed a weak positive correlation with maternal age (r = 0.249, *p* = 0.121). Apgar scores were not correlated with GFR.

## 4. Discussion

In this study, we evaluated the clinical outcomes of pregnant women with symptomatic gestational hydronephrosis who were managed with either ureteral double-J stent placement or conservative therapy. Our findings indicate that while ureteral stents may offer symptom relief in select patients, they do not significantly improve renal function or obstetric outcomes. This suggests that the decision to intervene should be highly individualized, weighing the severity of symptoms, gestational timing, and overall risk profile.

Gestational hydronephrosis is widely regarded as a physiological phenomenon caused by a combination of hormonal influences—particularly progesterone-induced smooth muscle relaxation—and mechanical compression from the enlarging uterus, especially on the right ureter [13,14]. Although often asymptomatic, GH may become clinically significant when it leads to flank pain, recurrent urinary tract infections, or impaired renal function. Current management strategies remain heterogeneous, ranging from conservative monitoring to invasive intervention with ureteral stents. In line with prior studies [1,3,15], our results support conservative treatment as the initial approach in mild to moderate cases.

Our analysis revealed no significant improvement in renal function parameters (serum creatinine and GFR) following stent placement, corroborating previous reports suggesting that GH rarely leads to meaningful renal deterioration in the absence of additional pathology [16,17,18]. Notably, we observed that patients in the stent group had a more advanced gestational age at the time of intervention (*p* = 0.045), which may reflect an increased symptom burden and ureteral compression as pregnancy progresses.

Inflammatory markers (CRP, ESR, and WBC count) were not significantly different between the groups. While some studies suggest that GH may be associated with low-grade inflammation, our results align with those reporting minimal systemic inflammatory involvement, regardless of treatment modality [19,20]. This implies that stent placement may not significantly alter the underlying inflammatory milieu.

In terms of obstetric outcomes, no significant differences were found in birth weight or Apgar scores. These findings are reassuring and consistent with literature indicating that stent placement, when required, does not pose additional fetal risk. However, a notable and unexpected finding was the significantly higher incidence of gestational diabetes mellitus (GDM) in the non-stent group (*p* = 0.042). Although the mechanism underlying this association is unclear, it raises interesting hypotheses. It is possible that increased hydration protocols used in conservative treatment may impact glucose metabolism or maternal stress responses. Alternatively, this association may be incidental due to sample size limitations and warrants further prospective investigation [21,22].

Postpartum complications such as infection and stent revision were rare, with similar rates between the groups, which is consistent with previous reports suggesting that stenting does not increase maternal morbidity in this context [23,24,25]. Nevertheless, even in the absence of infection, stents may contribute to discomfort, hematuria, and dysuria, emphasizing the need for appropriate patient counseling and follow-up.

Our study provides a comprehensive institutional snapshot of GH management strategies. While some earlier studies reported favorable outcomes with stent placement [26], others noted higher complication rates and no clear benefit over conservative approaches [5,27]. Our findings support the latter view and highlight that in the absence of overt renal compromise, conservative management remains appropriate.

It is increasingly evident that a risk-adapted, symptom-guided approach is the most rational strategy for GH management. Most cases are self-limiting and resolve spontaneously postpartum; thus, invasive interventions should be reserved for those with unrelenting symptoms or documented renal impairment. Emerging tools such as Doppler ultrasonography and renal resistive index measurement may further enhance patient stratification and decision-making in future practice [1,28].

This study is not without limitations. First, its retrospective design inherently restricts causal inference and generalizability. Second, the sample size is relatively small, although G*Power analysis confirmed statistical adequacy. Third, our follow-up period was limited to the immediate postpartum phase, precluding any conclusions regarding long-term renal function or stent-related complications. Furthermore, the lack of standardized quality-of-life or symptom burden assessment tools limited the patient-centered evaluation of stent versus conservative strategies. These gaps underscore the need for well-designed, prospective studies with larger cohorts and validated outcome metrics.

## 5. Conclusions

In conclusion, ureteral stent placement in pregnant patients with symptomatic gestational hydronephrosis does not significantly improve renal function or obstetric outcomes but may serve as an effective option for symptom control in selected cases. While stent use does not appear to increase maternal or neonatal risk, the observed association between conservative treatment and a higher GDM incidence warrants further investigation. Given the absence of standardized treatment guidelines, future research should focus on developing stratified clinical decision-making algorithms incorporating symptom severity, renal function indicators, and patient preferences to guide optimal management.

## Figures and Tables

**Table 1:** Comparison of Clinical and Obstetric Outcomes Between Groups.

Parameter	Stent Group (Mean ± SD/%)	Non-Stent Group (Mean ± SD/%)	*p*-Value
Age (years)	32.3 ± 5.6	32.5 ± 4.9	0.754
Gestational Age (weeks)	29.1 ± 3.2	27.1 ± 3.5	0.045
Creatinine (mg/dL)	0.98 ± 0.3	1.02 ± 0.4	0.612
GFR (mL/min)	95.2 ± 14.6	91.8 ± 15.2	0.289
CRP (mg/L)	4.2 ± 2.5	5.1 ± 3.1	0.402
ESR (mm/h)	19.3 ± 8.5	21.7 ± 9.2	0.245
Proteinuria (%)	25%	15%	0.378
Microscopic Hematuria (%)	35%	25%	0.291
Systolic BP (mmHg)	125.8 ± 9.6	123.5 ± 10.2	0.458
Diastolic BP (mmHg)	79.3 ± 10.2	77.6 ± 9.8	0.619
Birth Weight (g)	2949.2 ± 382.4	3069.4 ± 400.2	0.089
Apgar Score at 1 min	7.1 ± 1.2	7.3 ± 1.1	0.254
Apgar Score at 5 min	8.0 ± 0.9	8.0 ± 1.0	0.437
Gestational Diabetes (%)	5%	20%	0.042
Preeclampsia (%)	20%	15%	0.285
Postpartum Infection (%)	15%	10%	0.489
Ureteral Stent Revision (%)	5%	0%	0.073

**Table 2:** Logistic Regression Analysis for Predictors of Stent Placement.

Parameter	Coefficient	Odds Ratio	*p*-Value
Age	0.021	1.02	0.758
Birth Weight	−0.007	1.00	0.192
WBC Count	0.064	1.07	0.323
Hemoglobin	−0.050	0.95	0.156
Apgar Score at 1 min	−0.069	0.93	0.227

**Table 3:** Correlation Analysis Between Selected Clinical Parameters.

Parameter	Correlation With	Correlation Coefficient	*p*-Value
Gestational Age	WBC Count	−0.108	0.506
Creatinine	Maternal Age	0.249	0.121
Apgar Score at 1 min	GFR	−0.067	0.681

## Data Availability

The raw data supporting the conclusions of this article will be made available by the authors on request.
